# Effects of woodland slope on heavy metal migration via surface runoff, interflow, and sediments in sewage sludge application

**DOI:** 10.1038/s41598-024-64163-9

**Published:** 2024-06-12

**Authors:** Lihua Xian, Dehao Lu, Yuantong Yang, Jiayi Feng, Jianbo Fang, Douglass F. Jacobs, Daoming Wu, Shucai Zeng

**Affiliations:** 1 College of Forestry & Landscape Architecture, South China Agricultural University, Guangzhou, China; 2 Guangdong Eco-Engineering Polytechnic, Guangzhou, Guangdong China; 3 Department of Forestry and Natural Resources, Purdue University, West Lafayette, USA

**Keywords:** Sludge utilization, Soil contamination, Forest soils, Surface runoff, Rainfall, Forest ecology, Forestry

## Abstract

Sewage sludge (SS) application to forest plantation soils as a fertilizer and/or soil amendment is increasingly adopted in plantation forest management. However, the potential risks of SS-derived heavy metals (HMs) remain a concern. Many factors, including woodland slope may affect the risks, but the understanding of this issue is limited. This research evaluated the HMs migration via surface runoff, interflow, and sediments when SS was applied in woodlands of varying slopes. We conducted indoor rainfall simulations and natural rainfall experiments to clarify the effect of slope on the migration of HMs via runoff (including surface and interflow) and sediments. In the simulated rainfall experiment, HMs lost via sediments increased by 9.79–27.28% when the slope increased from 5° to 25°. However, in the natural rainfall experiment, when the slope of forested land increased from 7° to 23°, HMs lost via surface runoff increased by 2.38% to 6.13%. These results indciate that the surface runoff water on a high slope (25°) posed high water quality pollution risks. The migration of HMs via surface runoff water or interflow increased as the steepness of the slope increased. The total migration of Cu, Zn, Pb, Ni, Cr and Cd via sediment greatly exceeded that via surface runoff and interflow. Particles ≤ 0.05 mm contributed the most to the ecological risks posed by sediments. Cd was the main source of potential ecological risks in sediments under both experimental conditions.

## Introduction

Urbanization and global industrialization have led to increases in the production of sewage sludge (SS). SS production has thus become a major environmental issue^1,2^. Approximately 120 million tons of SS were generated by the major economies worldwide in 2019^3^, and the amount of SS generated by these economies is projected to be between 150 and 200 million tons in 2025. The composition of SS is highly complex, and previous studies have shown that SS contains various potentially harmful substances, including heavy metals (HMs), organic compounds, pathogens, and pharmaceutical residues^4^. In light of global limitations in the disposal capacity of SS, ensuring the safety and efficiency of the recycling and treatment of SS has become a major focus of research in the environmental science field.

SS has been applied as a fertilizer to woodlands, representing a sustainable and effective treatment for recycling the abundant organic matter and nutrients in SS^5–8^. As most forest products do not directly enter the human food chain, SS can be applied to forest land to enhance soil fertility and tree growth. However, the risk of contamination with HM elements, such as copper (Cu), zinc (Zn), lead (Pb), cadmium (Cd), and nickel (Ni) in SS, poses a major challenge for its use in woodlands at large scales^9–12^. HMs in SS may migrate through surface runoff to neighboring woodlands, where they are absorbed by the soil and accumulate, and this can have deleterious effects on plants, animals, and forest ecosystems^13,14^. Rainfall can facilitate the infiltration of HMs from SS into the subsoil, which affects groundwater quality and can pollute neighboring water resources^15–17^. However, the application of SS to woodlands enhances the physicochemical properties of the soil, which reduces the risk of HM migration in runoff^18^. The high content of organic matter in SS improves soil structure and increases the stability and erosion resistance of soil aggregates, which promotes the immobilization of HMs in the soil and reduces their susceptibility to transport^19^. Galdos et al.^20^ found that the application of SS increases the concentration of HMs in surface runoff and sediments and the migration of most HMs mainly occurs through sediments, indicating that the risk of HM migration via sediments is particularly high. Comprehensive assessments of the safety and sustainability of applying SS to woodlands are thus critically important.

Recent studies have shown that slope has a major effect on the risks of HM migration via runoff and sediment following the application of SS. Several factors contribute to the effect of slope on HM migration via these pathways. First, soil HMs are mainly in dissolved and particulate forms when they migrate with runoff during rainfall^21^. During this process, increases in slope slow the release of soil HMs via changes in the impact angle of raindrops and gravity, which can alter the form of HMs^22^. In SS, HMs are mainly in a granular state; however, during rainfall, changes in slope might promote the conversion of HMs into the dissolved state, which alters the total amount of HM migration and the relative contributions of different pathways (surface runoff, interflow, and sediments) to HM migration. Second, given that runoff and sediment are important carriers of HMs, runoff and sediment yield are key factors affecting the migration of HMs. Some studies have indicated that the residence time of precipitation on slopes decreases as the slope increases, which leads to decreases in the retention time of water in the soil, increases in the surface runoff on the slope^23^, and decreases in the yield of interflow^24^. However, other studies have shown that runoff volume reaches a critical threshold as the slope increases; beyond this threshold, runoff volume decreases or remains constant^25^. Furthermore, several studies have found that changes in slope are the main driver of the flushing of sediments by rainfall runoff^26^. High slopes can increase erosion by promoting soil stripping or mitigating the protective effect of SS on the soil surface. In the early stages of flow production, cumulative sediment migration increases with cumulative runoff, and transport-limited erosion is the dominant sediment erosion process. However, segregation-limited erosion becomes more prevalent in subsequent rainfall events^27^.

HMs can be transported via surface runoff, interflow, and sediment. However, only a few studies evaluating soil erosion and nutrient loss from agricultural fields have examined the relative contributions of all three of these pathways to the transport of HMs^28,29^. Additionally, few studies have clarified the effects of slope on the migration of HMs via surface runoff, interflow, and sediments during the application of SS to woodlands. Estimates of the magnitude of the potential ecological risks of HM migration via runoff might vary depending on the methodology of rainfall experiments. The effects of slope on the migration of HMs in runoff have generally been estimated via simulated rainfall experiments or natural rainfall experiments in the field^30,31^. However, whether, and to what extent, the risks of HM migration via runoff and sediment estimated using these two experimental approaches varies remains unclear. Here, we studied the migration of the HMs in SS via surface runoff (including surface flow and loam center flow), interflow, and sediment on different slopes simultaneously using an indoor simulated rainfall experiment and natural rainfall experiment in the field. The indoor simulated rainfall experiment was conducted in a homemade steel tank, and the natural rainfall experiment was conducted in a runoff plot in a *Eucalyptus* woodland. We then evaluated (1) the effects of slope on runoff water and sediment yield, (2) differences in the migration patterns of HMs in surface runoff water, interflow, and sediments on different slopes, and (3) the potential ecological risks of HM migration associated with the application of SS to woodland. Overall, the main aim of this study was to identify the optimal slope for enhancing the efficacy and safety of SS application.

## Results

### Runoff and sediment yield in simulated rainfall and natural rainfall experiments

#### Runoff and sediment yield in the simulated rainfall experiment

**Figure 1:**
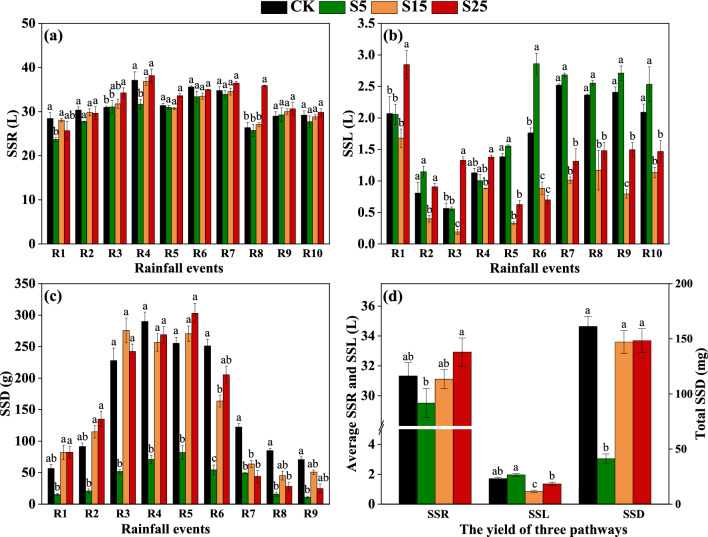
Surface runoff yield (SSR, L) (**a**), interflow yield (SSL, L) (**b**), and sediment yield (SSD,·g) (**c**) in each simulated rainfall event (R1–R10) (sediment was collected only in R1–R9). Total SSR (L), total SSL (L), and total SSD (g) of all 10 or 9 simulated rainfall events (CR10, CR9) (**d**). The data in the figure are the average of three replicates, and different letters indicate significant differences between treatments for the same simulated rainfall event. The threshold for statistical significance was *p* < 0.05. CK: Slope 15° + no SS application, S5: Slope 5° + SS application, S15: slope 15° + SS application, and S25: slope 25° + SS application.

Slope had a major effect on surface runoff, interflow, and sediment yield. Surface runoff yield in the simulated rainfall events was highest in the S25 treatment, followed by the CK, S15, and S5 treatments. Surface runoff yield for all rainfall events in the S15 and CK treatments did not significantly differ (Fig. 1a). The mean interflow yield was 132.17% and 45.14% higher in the S5 treatment than in the S15 and S25 treatments, respectively (Fig. 1b). The interflow yield was significantly higher in the S5 treatment than in the S15 and S25 treatments from the fifth (R5) to the tenth (R10) rainfall event. The sediment yield was significantly lower in the S5 treatment than in the other treatments for all rainfall events (Fig. 1c). Overall, the average yield of surface runoff increased as the slope increased. The average interflow yield was highest in the S5 treatment, which was significantly higher than in the S15 and S25 treatments. The average sediment yield in the S5 was significantly lower than in the other treatments (Fig. 1d).

**Figure 2:**
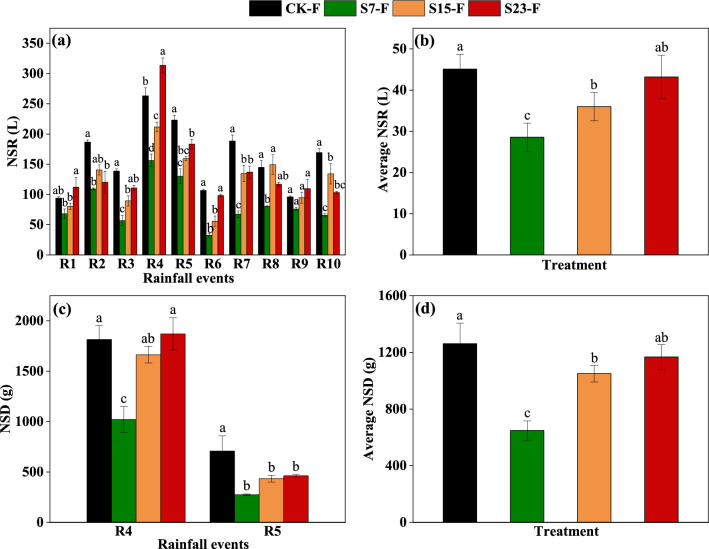
Surface runoff yield (NSR, L) (**a**), interflow yield (NSR, L) (**b**), and sediment yield (NSD, g) (**c**) for each natural rainfall event (R1–R10) (sediment was collected only in R4 and R5). Total NSR (L) and total NSD (g) of the 10 or 2 natural rainfall events (CR10, CR2) (**d**) The data in the figure are the average of three replicates, and different letters indicate significant differences between treatments for the same natural rainfall event. The threshold for statistical significance was p < 0.05. CK: Slope 15° + no SS application, S5: Slope 5° + SS application, S15: slope 15° + SS application, and S25: slope 25° + SS application.

Runoff and sediment yield in the natural rainfall experiment. The average surface runoff yield in the natural rainfall experiment increased as the slope increased, and the surface runoff yield in S15-F was significantly lower than in the CK-F for R3, R4, R5, R6, R7, and R10 (Fig. 2a). The sediment yield increased with the slope for both R4 and R5. The sediment yield for R5 was significantly lower in the S15-F treatment than in the CK-F treatment. No sediment was collected in the R1–R3 and R6–R10 rainfall events (Fig. 2c). The average surface runoff and sediment yield increased as the slope increased, with the mean surface runoff yield and sediment yield in S15-F 25.36% and 20.25% lower than CK-F, respectively, and these differences were significant (Fig. 2b,d).

### Cumulative amount of HM migration and the relative contributions of different pathways to HM migration in the simulated rainfall and natural rainfall experiments

**Figure 3:**
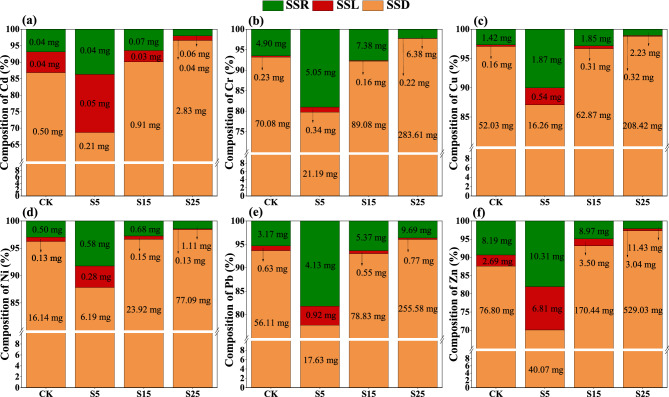
(**a**–**f**) are the cumulative migration amount (mg) and proportion (%) of Cd, Cr, Cu, Ni, Pb, and Zn with surface runoff (SSR), interflow (SSL), and sediment (SSD) in simulated rainfall events, respectively. CK: slope 15° + no SS application, S5: slope 5° + SS application, S15: slope 15° + SS application, and S25: slope 25° + SS application.

Cumulative amount of HM migration and the relative contributions of different pathways to HM migration in the simulated rainfall experiment. The cumulative amount of HM migration via surface runoff, interflow, and sediment, as well as the relative contributions of the different pathways to HM migration, were affected by the slope (Fig. 3). The cumulative migration amount for each HM in the sediments gradually increased as the slope increased. When the slope increased from 5° to 25°, the loss of HMs in sediments increased by 9.79–27.28%, and the loss of HMs in surface runoff and interflow decreased by 5.47–16.80% and 1.11–16.25%, respectively. Sediment was the main route for the migration of Cd, Cr, Cu, Ni, Pb, and Zn, and migration of these HMs via sediment was an order of magnitude greater than that via surface runoff and interflow.

#### Cumulative amount of HM migration and the relative contributions of different pathways to the migration of HMs in the natural rainfall experiment

**Figure 4:**
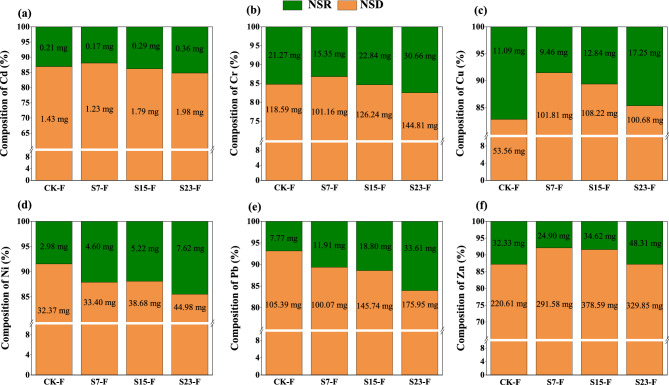
(**a**–**f**) show the cumulative migration amount (mg) and proportion (%) of Cd, Cr, Cu, Ni, Pb, and Zn via surface runoff (NSR) and sediment (NSD) in natural rainfall events, respectively. CK-F: slope 15° + no SS application, S7-F: slope 7° + SS application, S15: slope 15° + SS application, and S23: slope 23° + SS application.

Consistent with the results of the simulated rainfall experiment, the cumulative migration of HMs with surface runoff increased as the slope increased (Fig. 4). The proportion of cumulative amount of HM migration via sediment decreased with increasing slope, which was inconsistent with the results of the simulated rainfall experiment. Nevertheless, sediment was still the main pathway of Cd, Cr, Cu, Ni, Pb, and Zn migration, and the migration of these HMs via sediment was an order of magnitude greater than that via surface runoff. In each treatment, the proportion of HM migration via sediment was greater than 75%.

#### Comparison of the total migration of HMs on different slopes in the simulated rainfall and natural rainfall experiments

**Figure 5:**
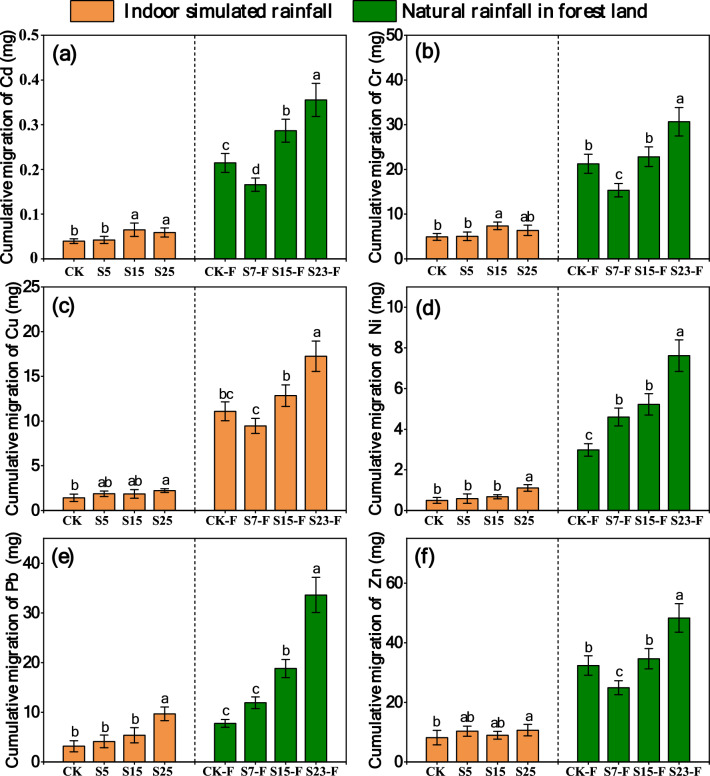
(**a**–**f**) show the total migration of Cd, Cr, Cu, Ni, Pb, and Zn via surface runoff for 10 simulated rainfall events and 10 forest natural rainfall events, respectively. CK: Slope 15° + no SS application, S5: Slope 5° + SS application, S15: Slope 15° + SS application, and S25: slope 25° + SS application (simulated rainfall experiment). CK-F: Slope 15° + no SS application, S7-F: slope 7° + SS application, S15-F: Slope 15° + SS application, and S23-F: slope 23° + SS application (natural rainfall experiment). The data in the figure are the average of three replicates, and different letters indicate significant differences between treatments (*p* < 0.05).

The total migration of HMs in surface runoff in the simulated rainfall and natural rainfall experiments varied with slope. Overall, the total migration of HMs in the natural rainfall experiment increased significantly as the slope increased, while the total amount of HMs migration in the simulated rainfall experiment varied with slope and HMs. The total migration of Ni, Pb, and Cu was similar in the simulated indoor rainfall and natural rainfall experiments, and the migration of these elements significantly increased as the slope increased (p < 0.05). The total migration of Cr and Cd first increased and then decreased as the slope increased, and the total migration of Zn first decreased and then increased as the slope increased in both the simulated rainfall and natural rainfall experiments (Fig. 5).

**Figure 6:**
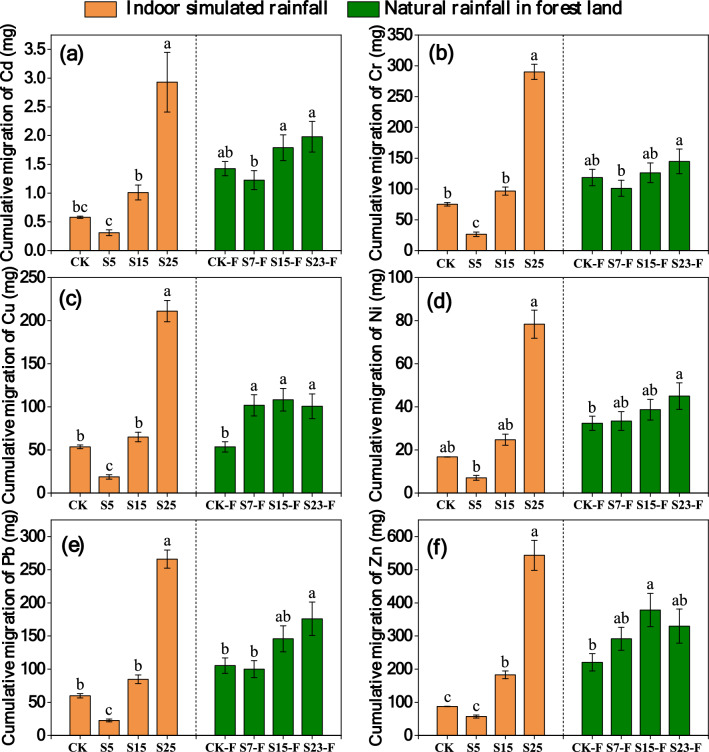
(**a**–**f**) show the total migration of Cd, Cr, Cu, Ni, Pb, and Zn via sediment for 10 simulated rainfall events and 10 forest natural rainfall events, respectively. CK-F: Slope 15° + no SS application, S7-F: Slope 7° + SS application, S15-F: Slope 15° + SS application, and S23-F: slope 23° + SS application (simulated rainfall experiment). CK-F: Slope 15° + no SS application, S7-F: slope 7° + SS application, S15-F: Slope 15° + SS application, S23-F: slope 23° + SS application (natural rainfall experiment). The data in the figure are the average of three replicates, and different letters indicate significant differences between treatments (*p* < 0.05).

The total migration of HMs via sediment increased significantly as the slope increased in the simulated indoor rainfall experiment. The total migration of HMs varied with slope in the natural rainfall experiment, and variation in the amount of HMs with slope differed among HMs. The total migration of Cd, Cr, Ni, and Pb increased as the slope increased in the natural rainfall and indoor simulated rainfall experiments. However, the total migration of Cu and Zn first increased and then decreased as the slope increased, and this was inconsistent with the results in the indoor simulated rainfall experiment (Fig. 6).

### Potential ecological risks of HM migration via runoff and sediment in the simulated rainfall and natural rainfall experiments

#### Potential ecological risks of HM migration via runoff and sediment in the indoor simulated rainfall experiment

**Figure 7:**
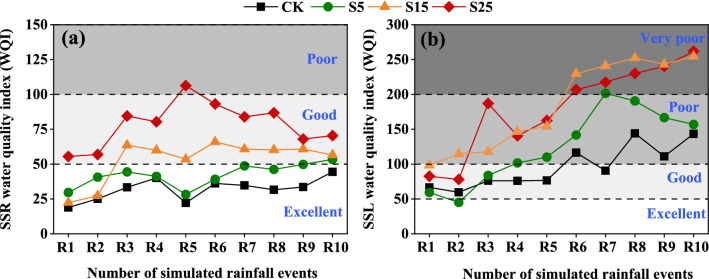
(**a**, **b**) show WQI values of surface runoff (SSR) and interflow (SSL) in simulated rainfall events, respectively. CK: Slope 15° + no SS application, S5: Slope 5° + SS application, S15: Slope 15° + SS application, and S25: slope 25° + SS application. WQI < 50, 50 ≤ WQI < 100, 100 ≤ WQI < 200, 200 ≤ WQI < 300, and WQI ≥ 300 correspond to excellent, good, poor, extremely poor, and unfit for drinking, respectively.

The WQI was used to evaluate changes in the water quality of surface runoff and interflow during 10 simulated rainfall events (Fig. 7). In the 10 rainfall events, the water quality of the surface runoff in the CK and S5 treatments was "excellent." In R1–R2, the surface runoff water quality in the S15 treatment was "excellent", and it changed from "excellent" to "good" as the number of simulated rainfall events increased. In the S25 treatment, the surface runoff water quality was "poor" for R4, but "good" for the other nine rainfall events (Fig. 7a). The interflow water quality in CK and S5 changed from "good" to "poor" and that in S15 and S25 changed from "good" to "very poor" as number of simulated rainfall events increased (Fig. 7b).

The potential ecological risk assessment revealed (Table 1) that the *E*_*r*_ of Cd in S15 and S25 was "considerable" in sediments with particle sizes > 0.05 mm and "high" when particle sizes were ≤ 0.05 mm. The *E*_*r*_ of Cd in the S5 treatment was "high" when particle sizes were ≤ 0.05 mm and "low" in sediments with particle sizes > 0.05 mm. The potential ecological risks of Cd for the four sediment particle sizes were 74.05–112.85% higher in the S15 treatment than in the CK. The *E*_*r*_ of the other five HMs for the four particle sizes in the different treatments was "low."

**Table 1:** The potential ecological risks of HMs in sediment of the four particle sizes on different slopes in the simulated rainfall experiment.

Particle sizes (mm)	Treatment	Potential ecological risk coefficients (*E*_*r*_) of single elements						Potential ecological risk index (*RI*) of multiple elements	Risk level
		Cd	Cr	Cu	Ni	Pb	Zn		
> 1	CK	62.29	0.87	4.30	1.68	2.54	0.52	72.20	Low
	S5	23.53	0.25	1.30	0.47	1.17	0.47	27.19	Low
	S15	110.72	1.23	6.13	2.45	4.95	1.86	127.34	Low
	S25	107.89	1.07	5.12	2.05	4.42	1.65	122.20	Low
0.25–1	CK	56.49	0.91	4.41	1.68	2.91	0.66	67.06	Low
	S5	31.38	0.31	1.47	0.59	1.11	0.33	35.19	Low
	S15	120.24	1.23	5.29	2.34	4.58	1.43	135.11	Low
	S25	95.11	1.00	4.72	1.98	3.99	1.14	107.94	Low
0.05–0.25	CK	84.08	1.17	6.61	2.46	3.40	0.68	98.40	Low
	S5	35.48	0.38	2.02	0.86	1.10	0.33	40.17	Low
	S15	174.81	1.72	8.92	4.32	5.15	1.48	196.40	Moderate
	S25	133.88	1.35	7.70	3.39	3.67	1.24	151.23	Moderate
≤ 0.05	CK	104.03	1.33	9.07	3.77	3.86	0.76	122.82	Low
	S5	177.02	1.70	10.32	5.01	4.79	1.62	200.46	Moderate
	S15	181.06	1.72	10.38	4.97	4.82	1.63	204.58	Moderate
	S25	177.13	1.72	10.99	5.00	4.98	1.65	201.47	Moderate

Data in the table are the average of three replicates. CK: Slope 15° + no SS application, S5: Slope 5° + SS application, S15: Slope 15° + SS application, and S25: slope 25° + SS application. *E*_*r*_ was classified as follows: low < 40; 40 ≤ moderate < 80; 80 ≤ considerable < 160; and 160 ≤ high < 320. *RI* was classified as follows: low risk < 150; 150 ≤ moderate risks < 300; 300 ≤ considerable risks < 600; and high risk ≥ 600.

The *RI* value in the sediment depends on the slope and particle size of the sediment (Table 1). In the simulated rainfall experiment, the *RI* values of the sediments for the four particle sizes were highest in the S15 treatment. The *RI* values of sediments were highest and lowest in different treatments for particle sizes ≤ 0.05 mm and > 1 mm, respectively. *RI* values of sediments increased as the particle size decreased. Generally, when the sediment particle size was ≤ 0.25 mm, the *RI* was "low"; when the sediment particle size was 0.05 ~ 0.25 mm, the *RI* of the CK and S5 treatment was "low", and the *RI* of the S15 and S25 treatments was " moderate"; when the sediment particle size was ≤ 0.05 mm, the *RI* of the CK was "low." The *RI* of the other three treatments was "moderate." The potential ecological risks of each treatment mainly stemmed from Cd (Table 1).

#### Potential ecological risks of HM migration via runoff and sediment in the natural rainfall experiment

**Figure 8:**
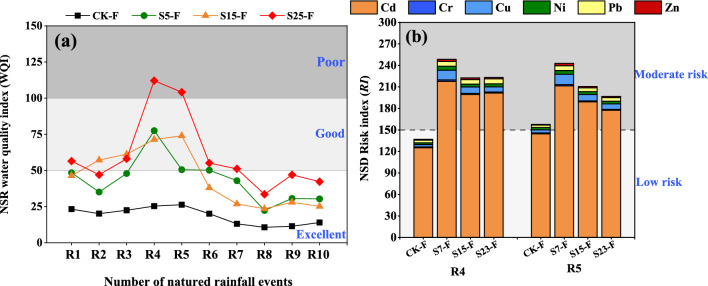
WQI value of surface runoff (NSR) in 10 natural rainfall events (**a**). *RI* value of sediment (NSD) for two natural rainfall events in forest land (**b**). CK-F: Slope 15° + no SS application, S7-F: slope 7° + SS application, S15-F: slope 15° + SS application, and S23: slope 23° + SS application. *RI* can be categorized as follows: low risk < 150; 150 ≤ moderate risks < 300; 300 ≤ considerable risks < 600; and high risk ≥ 600. WQI < 50, 50 ≤ WQI < 100, 100 ≤ WQI < 200, 200 ≤ WQI < 300, and WQI ≥ 300 correspond to excellent, good, poor, extremely poor, and unfit for drinking, respectively.

No significant differences in the WQI of the surface runoff of each treatment were observed for R1–R3. In R4, the WQI of surface runoff was 61.82%, 16.57%, and 92.92% higher in the S7-F, S15-F, and S23-F treatments than in the R3 treatment, respectively. The WQI of each treatment decreased from R5 to R10, and the water quality was "excellent" for R8–R10 (Fig. 8a). Sediment was collected during only R4 and R5. The potential ecological risks of the three treatments with SS application were significantly higher than CK-F. Consistent with the results of the simulated rainfall experiment, Cd was the main source of potential ecological risks in sediment in the natural rainfall experiment, and the E_r_ value for Cd was significantly higher than that for the other five HMs. The RI values of the sediments for R4 and R5 decreased as the slope increased (Fig. 8b).

## Discussion

The use of SS as fertilizer for forest soil is a promising SS treatment method. However, HMs can migrate to the external environment via surface runoff, interflow, and sediment following SS application. The risks of HM migration might be particularly high in hot and rainy southern forest regions. Evaluating the risks of HM migration via these different pathways is critically important for ensuring the safety of SS application^32,33^. SS application to forest land might alter the erosion effect of rainfall on soil, and the amount of HM migration via surface runoff, interflow, and sediment varies with slope^20,24^ The results of the indoor simulated rainfall experiments showed that the surface runoff yield increased as the slope increased following SS application. We also found that changes in surface runoff and sediment yield with slope were similar in the natural rainfall and indoor simulated rainfall experiments. This indicates that slope has a major effect on the transport of runoff, even in natural woodlands. Season is one of the main factors affecting surface runoff and sediment yield in natural rainfall experiments in woodlands. In the natural rainfall experiment, 70% of the rainfall events occurred in the summer (from May to August). Furthermore, patterns of variation in rainfall and surface runoff were similar for the 10 natural rainfall events. These findings suggest that surface runoff yield in natural systems is strongly affected by season and the amount of rainfall. We found that the interflow yield was highest in the S5 and lowest in S15 in the simulated rainfall experiment. Zhang et al.^25^ showed that permeability decreases as the slope increases in an indoor simulated rainfall experiment. Other studies have shown that the soil infiltration rate decreases significantly as the slope increases when the slope is less than 18°. Slope has a weak effect on the infiltration when it is greater than 18°^34^. This is consistent with the findings of Wu et al.^23^ showing that there is a critical slope which causes the runoff to reach the maximum. The relationship between slope and infiltration changes as the slope increases^24^. When the slope is 15°, the splashing effect of rainfall on topsoil has a major effect on the physical properties of soil, alters the roughness of topsoil, promotes soil compaction, and reduces the interflow yield. This might also explain why the interflow yield was significantly lower in the S15 treatment than in the S5 and S25 treatments.

Sediment production was lower in the S15 treatment than in the CK in the simulated rainfall experiment. This stems from the fact that the large particles prevent the erosion of SS by runoff because they form a protective layer that prevents the erosion of soil particles by precipitation^35^. The sediment yield increased as the slope increased. This might be explained by the small area of rainfall in our experiment; furthermore, the surface water flow velocity and the shear stress increase as the slope increases^36^. However, the retention time of surface water on the slope decreases as the slope increases, which weakens the protective effect of SS on the soil and increases the strength of the splashing effect of rainfall on the topsoil^37^; this results in the activation and transport of more soil particles. However, the sediment yield was 255.92% and 258.82% lower in the S5 treatment than in the S15 and S25 treatments, respectively. Wang et al.^38^ found that surface runoff flow is unable to carry or separate sediments with larger grain sizes on low slopes, and this results in a drastic decrease in sediment yield. This can be explained by the fact that large particle sizes experience greater resistance in raindrops and surface flow, which inhibits their removal. Specifically, the sediment yield first increased and then decreased, suggesting that transport-limited erosion was the dominant erosion process in the early rainfall events^39^. The effect of raindrops on flow transport might also contribute to the mechanism restricting sediment transport^27^. As the number of rainfall events increased, segregation-limited erosion became the dominant erosion process. These results are consistent with those of Ran et al. ^40^ and Shi et al.^27^. We found that patterns of variation in sediment and surface runoff yield were similar in the natural rainfall and simulated indoor rainfall experiments, and both sediment and surface runoff yield increased as the slope increased. Large sediment particles cannot be activated when the intensity of rainfall is low because of the weak driving force^38^.

Organic matter plays a pivotal role in the soil’s capacity to adsorb HMs^41^. Functional groups of organic matter, such as carboxylic (–COOH) and hydroxyl (–OH) groups, have the ability to form complexes with metal ions. We found that ROC and LOC were significantly and positively correlated with the total migration of most HMs, except for TCd, which showed no significant correlation with ROC (Table S1). This discrepancy can be attributed differences among HMs in their ability to bind organic matter. Relevant studies show that Cd^2+^ has a low affinity for organic compounds. Cu^2+^ is more easily combined with organic matter^42,43^. Additionally, the distinct adsorption kinetics of Cu and Zn imply that Cu is more prone to forming complexes with organic matter, leading to slower adsorption rates and rapid release rates for Zn^43^, potentially explaining the higher migration rates of Zn in runoff. We also found a weak correlation between pH and total HM migration. Most studies have shown that under high pH conditions, HMs are easy to form complexes with iron, manganese and hydroxide, precipitate or migrate with the adsorption of sediment particles. Under low pH conditions, they are more likely to be desorbed and dissolved in soil solution, enter and migrate with surface runoff^44,45^. However, these studies mainly discussed the effect of pH on the metal form or the migration of HMs in different pathways, and did not discuss the change of the total amount of HMs in surface runoff, interflow and sediment under varying pH. It is a well-established fact that pH and EC are negatively correlated^44^. When soil permeability is high, the EC value also increases. During this time, the oxidation reaction is the primary process, resulting in a decrease in the H^+^ content, an increase in negative charge, and the adsorption of cations. When EC is low, soil permeability is poor. As a result, the mobility of cations is enhanced. This may also be the reason why TCr, TNi and TZn are significantly correlated with EC, while TCd, TCu and TPb are not significantly correlated with EC. Relevant studies have shown that the adsorption and desorption behavior of HMs in soil varies according to their different properties^46–48^. Zn and Ni mainly exist in the reducible component, Cu mainly exists in the oxidizable component, and Pb mainly exists in the residual component^49^. In addition, the migration of Cr and Pb through intercurrent is significantly lower than that through surface runoff. This may be related to the fraction distribution of Cr and Pb in SS, because the more stable metals in SS are difficult to remove by rainfall.

HMs in surface soils are mainly present in granular form during erosion, and the amount of migration is positively related to the slope^37^. As the slope increased, more sediment particles were entrained and washed away in the runoff, which led to an increase in the cumulative migration of HMs in the sediments. The cumulative amount of HM migration in the sediments accounted for more than 65% of the total amount of HM migration, indicating that sediment was the main pathway of HM migration; this is consistent with the findings of Galdos et al.^20^ and Huang et al.^50^. Although the total amount of each HM transported in both pathways (surface runoff and sediment) increased as the slope increased, the percentage of each HM in sediment and surface runoff decreased and increased, respectively; this finding was inconsistent with the results of the indoor simulated rainfall experiment. Rainfall events in forests typically last for several hours or even days; consequently, the surface soil in the natural rainfall experiment could have been saturated, which would increase the release of HMs via surface runoff. In addition, the splashing of raindrops has been shown to increase the dispersion and transport of soil particles by breaking up and moving aggregates and disrupting soil structure^51^. The presence of a forest canopy in forested areas mitigates the erosion of soil particles by the splashing of raindrops, which slows the transport of HMs via sediments.

We found that the risks of HM contamination in surface runoff water were higher when SS was applied compared with the CK; this is consistent with the results of most studies^13^. Furthermore, the water quality in surface runoff from the CK and S5 treatment was "excellent" during the 10 rainfall events, and there was virtually no risk of contamination in surface runoff on lower slopes. The WQI in the S15 and S25 treatments was "good" for most rainfall events, and only poor for the fourth rainfall event in the S25 treatment. This suggests that the risk of surface runoff pollution increases as the slope increases; overall, there was no risk of surface runoff water pollution. No significant change was observed in the risk of surface runoff water pollution as the number of rainfall events increased. Previous studies have shown that the application of sludge will lead to HMS pollution levels in farmland runoff exceeding Class IV water quality standards of China's Surface Water Environmental Quality Standards^20^. This difference might stem from variation in the soil and SS used in the different experiments. Another study has shown that the risk of HM transport is significantly increased in sandy soils^52^, and the risk of HM transport is reduced in lateritic soils rich in iron and aluminium oxides. We found that the pollution risk of surface runoff increased as the slope increased for 10 natural rainfall events, and the WQI was "poor" only in R4 and R5 of the S25 treatment; this was consistent with the results of the indoor simulated rainfall experiment.

Sediment was the main pathway of HM transport, and the magnitude of HM loss was an order of magnitude greater via sediment than via surface runoff and interflow. Previous studies have shown that the adsorption/desorption capacities of aggregates might vary for different particle sizes because of differences in soil physicochemical properties^50^. The *E*_*r*_ values of all HM elements were “low,” with the exception of that for Cd. In the indoor simulated rainfall experiment, the contribution of Cd to *RI* was 75% or more in all treatments. Other studies have also found Cd to be the main element contributing to ecological risks, and this might stem from the fact that it has the highest toxicity response factor among all metals tested^1,2,53^. The risk of sediment contamination was highest for two particle sizes: 0.05–0.25 mm and ≤ 0.05 mm. Previous studies have shown that fine particles typically have a higher surface area and pore volume compared with large particles, and this promotes the strong adsorption of HMs. Fine particles can be easily mobilized by water flow, and the transport of larger particles (> 0.25 mm) can only be initiated at higher flow rates^54^. Nevertheless, sediment was the main pathway for the migration of Cd, Cr, Cu, Ni, Pb, and Zn, and the migration of these HMs via sediment was an order of magnitude greater than that via surface runoff. Under laboratory conditions, predictions of HM transport from simulated rainfall experiments can lead to a range of conclusions based on the assumptions made^55^. However, the environmental complexity and variability of forests cannot be easily accounted for in indoor experiments. The results of the simulated and natural rainfall experiments were not completely consistent for different slopes. Thus, achieving an improved understanding of variation in HMs and their bioavailability in woodlands will require comparisons of the results of laboratory and field studies; these studies will also enhance our ability to investigate the mobility of HMs in natural woodlands using laboratory simulation experiments^56^.

## Conclusions

Slope had a significant effect on the runoff and sediment yield. Surface runoff and sediment yields increased as the slope increased in the simulated rainfall experiment. A similar pattern was observed in the natural rainfall experiment, as the surface runoff and sediment yield also increased with slope. Sediment was the main pathway for HM migration in all treatments, and more than 68.70% of the total migration of Cd, Cr, Cu, Pb, and Zn was via sediment. The application of suitable amounts of SS on lower slopes (5°) reduced runoff and soil erosion and did not significantly contribute to the risk of HM contamination in the simulated rainfall experiment. In the simulated rainfall experiment, the risk of contamination was only observed for sediments with a grain size ≤ 0.25 mm. Our findings revealed significant effects of slope on the risk of HM migration following SS application on forest land. However, more studies are needed to clarify the factors driving differences in the results of simulated and natural rainfall experiments, as well as their underlying mechanisms, in planted forests in the future due to the high complexity of the hydrological and surface soil conditions in the field.

## Methods

### SS and soil properties

SS was obtained from Guangzhou Water Purification Co., Ltd. in Guangdong Province, China. The water content of the treated SS was approximately 40%, and the HM content of the SS was high. SS was anaerobically composted for 60 d prior to its use. The SS was then air-dried, sieved through a 10 mm nylon sieve, and thoroughly mixed. The soil was collected from the Eucalyptus forest in Dalingshan Forest Park, Dongguan City, Guangdong Province, China (22°51′18.99ʺN, 113°45′22.44ʺE). The study area has a typical subtropical monsoon climate, with an average annual temperature of 23.3 °C and annual precipitation of 2042.6 mm. The soil is granite red soil and has a sandy loam texture (56.7% sand, 27.7% silt, and 15.6% clay). In November 2020, soil samples were collected from the 0–10 cm, 10–20 cm, and 20–30 cm layers. In the laboratory, soils were sampled using a ring knife, and the bulk weight was measured. The soils were air-dried at room temperature for two weeks, passed through a 10 mm nylon sieve, and mixed thoroughly. The soil and SS samples were then ground and sieved (< 0.15 mm), and the content of organic matter, Cd, chromium (Cr), Cu, Ni, Pb, and Zn, as well as the pH were measured (Table S2).

### Experimental design of the indoor rainfall simulations

The steel flumes comprise a steel tank and plastic external baffles. They were rectangular and had the following dimensions: 1.0 m × 0.3 m × 0.4 m. The length of the flumes of the soil tank was adjusted for different slopes. The internal part of the steel flumes comprised the soil sample receiving space with a base area of 0.3 m^2^ (1.0 m × 0.3 m) and a depth of 0.4 m. Plastic baffles (height of 0.3 m) were fitted around and on top of the steel flumes to prevent soil and water spillage. The bottom of the steel flumes was sealed with a PVC sheet, and the remaining small holes allowed the interflow to drain. The bottom was lined with quartz sand with a thickness of 3 cm, and it was washed with dilute nitric acid and lined with a 100-mesh screen. A V-shaped water-measuring weir was installed at the lower end of the rack to convey surface runoff water through a plastic pipe to a plastic collection bucket. The slope of the rack could be adjusted from 0° to 30°. To enhance the realism of the rainfall simulations, a 2 cm seepage space was installed at the bottom of the steel rack. The soil was raised 2 cm using a plastic mat. The interflow that drained from the soil and flumes was collected and placed at the bottom of the steel rack (Fig. S1)*.*

Simulated rainfall was used to evaluate the effect of slope on soil erosion and the migration of Cd, Cr, Cu, Ni, Pb, and Zn in surface runoff, interflow, and sediments. The experiment comprised four treatments, S5, S15, and S25 (in which SS was applied and the slope of the soil flume was 5°, 15°, and 25°, respectively) and CK (in which no SS was applied and the slope of the soil flume was 15°) (Table S3). Given that the slope altered the contact area between precipitation and the surface of soil troughs, 1.80 kg, 1.87 kg, and 1.98 kg of SS were applied in the S5, S15, and S25 treatments,which corresponds to 60 tons ha^−1^. The SS was thoroughly mixed with the surface soil in the 0–10 cm layer (Table S3). Each treatment has three replicates. The simulated rainfall water was municipal tap water. The chemical properties of the water were as follows: pH 7.4; Cd < 4 μg L^−1^; Cr < 4 μg L^−1^; Cu < 9 μg L^−1^; Pb < 0.07 μg L^−1^; and Zn < 1 μg L^−1^. The simulated rainfall experiment was performed in the simulated rainfall hall of the Red Soil Erosion and Flow Hydraulics Laboratory, Institute of Ecology, Environment and Soil, Guangdong Academy of Sciences, China. The rainfall system was comprised of a variable power pump (0–60 W with a water volume of 0–5 L min^−1^) and a water tank (1.2 m × 0.4 m × 0.12 m) with 150 holes (0.8 mm diameter) at the bottom. Water was pumped into the tank and dripped down through the holes at the bottom of the tank to form continuous and steady rainfall. The rainfall intensity was set to 120 mm h^−1^ to simulate heavy rainfall and severe soil erosion, which is consistent with the typical climatic characteristics of South China. Before the formal rainfall experiment, the rainfall intensity was adjusted to rainfall uniformity > 95%. A total of 10 simulated rainfall events were performed over a two-week interval between each rainfall event. The duration of each rainfall event was 60 min, and the total rainfall of the 10 simulations was equivalent to 3/4 of the rainfall in Guangzhou. The simulated rainfall experiment was initiated in March 2022 and ended in December 2022. The simulated rainfall experiment was carried out for two weeks, and there was a two-week interval between each rainfall event.

### Design of the natural rainfall experiment

*T*he study was conducted in a Eucalyptus plantation in Dalingshan Forest Park, Dongguan City, Guangdong Province, China. The Eucalyptus forest was planted in 1998 with a row spacing of 3 m × 2 m. The runoff plots were arranged according to the actual contours of the site, and the woodland slopes ranged from 5° to 30°. The runoff plots were sloping woodlands with similar characteristics except slope to ensure that rainfall conditions were consistent among plots. Each runoff plot has a horizontal projected area of 20 m^2^ (10 m × 2 m) in the direction of the slope. At the lower end of the runoff plot, there was a collection pond measuring 1 m × 1 m × 1 m, which was connected to the runoff plot. The pond was elevated 20 cm above the ground and covered with PVC plastic sheets to prevent rainwater from entering (Fig. S2).

The natural rainfall experiment was comprised of four treatments, S7-F, S15-F, and S23-F (SS was applied and their slopes were 7°, 15°, and 23°, respectively) and CK-F (in which the slope was 15°, and no SS was applied) (Table S3). Each treatment had three replicates. The amount of rainfall of the 10 natural rainfall events in the woodland and the amount of rainfall during the 48 h prior to sampling are shown in Fig. S3.

### Sample collection and analysis

In the indoor rainfall simulations, surface runoff and interflow were collected using two containers. Only surface runoff was collected in the natural rainfall experiment. When the surface runoff or interflow stopped, measurements were taken. After the collection containers were left to stand for 30 min, the suspensions were collected individually into 200 mL plastic bottles, and 1 mL of 1:10 nitric acid solution was added to inhibit microbial activity and prevent the precipitation of HM ions. The processed samples were stored at 4 °C. Using the wet sieving method, sediments precipitated in runoff collection containers during natural and simulated rainfall events were filtered and classified into four different types of agglomerates with different particle sizes: > 1 mm (large macro-aggregates), 1–0.25 mm (small macro-aggregates), 0.25–0.05 mm (large micro-aggregates), and < 0.05 mm (small micro-aggregates)^50,57^. Because the amount of sediment produced by the 10th rainfall was very small, the collection of particle size sediment samples was not carried out, and a total of 9 runoff sediment samples were collected in the simulative rainfall experiment. Sediment samples were only washed out and collected during the 4th and 5th natural rainfall experiment. Half of the agglomerates were dried, weighed, and milled separately, and the other half was mixed. Surface runoff, interflow, and sediment samples were digested using the triple-acid method (nitric-hydrofluoric-perchloric acid), and a plasma atomic emission spectrometer (Leeman Prodigy7 model) was used to determine the concentrations of Cd, Cr, Cu, Ni, Pb, and Zn.

### Calculation of indicators

Three indicators, surface runoff yield (L), interflow yield (L), and sediment yield (g), were used to evaluate the effects of different slopes on runoff and soil erosion. Cumulative lift volume (CLV) was used to evaluate the transport of HMs via three pathways, surface runoff, interflow, and sediment, for all rainfall events. CLV was calculated using Eqs. (1) and (2).

1 $$\text{LV }=\text{ SSR }(\text{NSR}/\text{SSL}/\text{NSL}/\text{SSD}/\text{NSD}) \times \text{ N}$$

2 $${\text{CLV}}_{n} =\text{ L}{\text{V}}_{1} +\text{ L}{\text{V}}_{2} \dots +{\text{LV}}_{n}$$

where N is the concentration of HMs in surface runoff, interflow, or sediments, which is multiplied by the surface runoff yield (SSR/NSR), interflow yield (SSL/NSL), or sediment yield (SSD/NSD), respectively, for each simulated or natural rainfall event. These values were used to calculate the amount of HM migration via the corresponding pathways (LV). CLV*n* was the cumulative migration of HMs in surface runoff, interflow, and sediments during 1st ~ nth rainfall events.

The water quality index (WQI) describes water quality via several water quality parameters^58^. We used WQI to evaluate the risk of HM pollution in surface runoff and interflow. WQI was calculated using the following formula:

3 $$WQI=\sum \left[{W}_{i}\times \left(\frac{{C}_{i}}{{S}_{i}}\right)\right]\times 100$$

In the WQI formula, ${W}_{i}$=${\text{w}}_{i}/\sum {w}_{i}$, where ${W}_{i}$ is the weight of each HM, and $\sum {w}_{i}$ is the sum of the weights of all HMs. The weights of Cd, Cr, Cu, Ni, Pb, and Zn were defined as 5, 5, 2, 4, 5, and 1, respectively, following a previous study^59^. ${C}_{i}$ corresponds to HM concentrations, and ${S}_{i}$ indicates the Chinese drinking water standard^12^. WQI was classified as excellent (WQI < 50), good (50 ≤ WQI < 100), poor (100 ≤ WQI < 200), very poor (200 ≤ WQI < 300), and unfit for drinking (WQI ≥ 300)^59^.

The potential ecological risk index (*RI*) was used to evaluate the ecological risk level of HMs in soil and evaluate the combined toxicity of HMs^60^. *RI* can be calculated using Eqs. (4) and (5);

4 $${E}_{i}={T}_{i}\times \frac{{C}_{i}}{{C}_{0}}$$

5 $$RI=\sum_{i=1}^{n}{E}_{i}$$

where ${E}_{i}$ is the risk factor for the given HM; ${T}_{i}$ is the toxicity response factor for the given pollutant (${T}_{i}$ for Cd, Cr, Cu, Ni, Pb, and Zn was defined as 30, 2, 5, 5, 5, and 1, respectively); ${C}_{i}$ is the concentration of each HM in the soil, and ${C}_{0}$ is the background concentration of HMs in the study area. Chu et al.^11^ found that the background concentrations of Cu, Zn, Pb, Cd, Cr, and Ni in Guangzhou were 28.7, 77.8, 57.6, 0.13, 87.0, and 23.5 mg kg^−1^, respectively. Values of ${E}_{i}$ were classified as follows: low < 40; 40 ≤ moderate < 80; 80 ≤ considerable < 160; and 160 ≤ high < 320. Values of *RI* were classified as follows: low risk < 150; 150 ≤ moderate risk < 300; 300 ≤ considerable risk < 600; and high risk ≥ 600.

### Statistical analysis

SPSS 19.0 (SPSS Inc., USA) was used to conduct all statistical analyses. One-way analysis of variance, followed by Duncan’s test, was used to evaluate the significance of differences among treatments (p < 0.05). Origin Pro 2019 software (Origin Lab Corporation, Northampton, MA) was used to make plots.

### Supplementary Information

Supplementary Information.

## Data Availability

The data that support the findings of this study is provided within the manuscript or supplementary information files.
