# Supplementary material for: Effects of woodland slope on heavy metal migration via surface runoff, interflow, and sediments in sewage sludge application
Source: Sci Rep. 2024 Jun 12;14:13468. doi: 10.1038/s41598-024-64163-9 (PMC11169265; doi:10.1038/s41598-024-64163-9)
Supplement: Supplementary file 1 — Supplementary Information. [file 41598_2024_64163_MOESM1_ESM.docx]

Effects of woodland slope on heavy metal migration via surface runoff, interflow, and sediments in sewage sludge application

Lihua Xian^1^，Dehao Lu^1^，Yuantong Yang^1^，Jiayi Feng^2^，Jianbo Fang^1^，Douglass F. Jacobs^3^，Daoming Wu^1^ & Shucai Zeng^1*^

^1^College of Forestry and Landscape Architecture, South China Agricultural University, Guangzhou, China. ^2^Guangdong Eco-Engineering Polytechnic, Guangzhou, Guangdong, China. ^3^Department of Forestry and Natural Resources, Purdue University, West Lafayette, USA.

Shucai Zeng

College of Forestry and Landscape Architecture, South China Agricultural University, 483 Wushan Road, Wushan Street, Guangzhou, 510642, PR China

E-mail: sczeng@scau.edu.cn

**Supporting information including 3 figures, 3 tables.**

**Figure S1.** Indoor rainmaker simulating rainfall.

**Figure S2.** Runoff plot design in the field.

**Figure S3.** Temporal distribution of natural rainfall in the forest land plots during the experimental period.

**Figure S1**

**
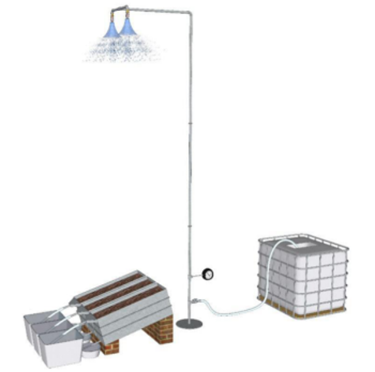
**

**Figure S2**


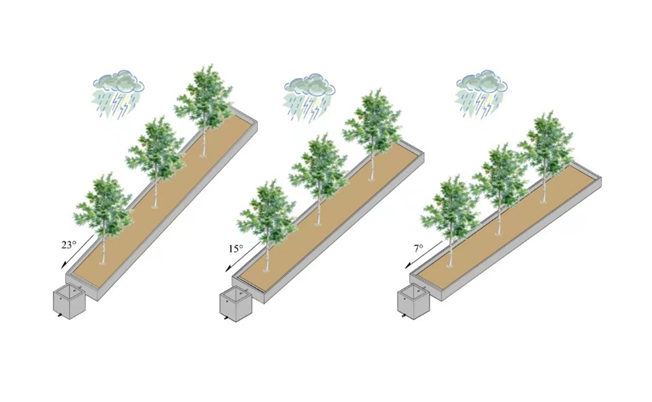


**Figure S3**

**Table S1.** The correlation between the physicochemical properties of runoff and sediment and the total amount of HMs.

| Index | TCr | TCd | TCu | TNi | TPb | TZn |
| --- | --- | --- | --- | --- | --- | --- |
| RpH | 0.087 | 0.023 | -0.024 | 0.004 | 0.041 | 0.155 |
| REC | 0.251** | 0.128 | 0.163 | 0.232* | 0.151 | 0.263** |
| SSR | 0.369*** | 0.377*** | 0.368*** | 0.377*** | 0.413*** | 0.342*** |
| ROC | 0.234* | 0.184 | 0.234* | 0.261** | 0.223* | 0.240* |
| RNN | 0.360*** | 0.252** | 0.295** | 0.383*** | 0.303** | 0.365*** |
| RAN | -0.174 | -0.19* | -0.196* | -0.159 | -0.236* | -0.223* |
| RSP | -0.024 | -0.105 | -0.078 | -0.014 | -0.009 | 0.007 |
| RTP | -0.121 | -0.194* | -0.195* | -0.166 | -0.174 | -0.089 |
| RTN | 0.428*** | 0.331*** | 0.354*** | 0.432*** | 0.330*** | 0.431*** |
| LpH | 0.045 | 0.038 | 0.072 | 0.082 | 0.063 | 0.019 |
| LEC | 0.185 | 0.091 | 0.039 | 0.090 | 0.134 | 0.249** |
| SSL | -0.430*** | -0.397*** | -0.408*** | -0.419*** | -0.419*** | -0.444*** |
| LOC | 0.387*** | 0.275** | 0.290** | 0.354*** | 0.297** | 0.393*** |
| LNN | 0.312** | 0.253** | 0.268** | 0.332*** | 0.306** | 0.316** |
| LAN | 0.223* | 0.149 | 0.162 | 0.204* | 0.146 | 0.250* |
| LSP | 0.064 | 0.004 | 0.063 | 0.106 | 0.090 | 0.062 |
| LTP | 0.188 | 0.119 | 0.122 | 0.151 | 0.145 | 0.215* |
| LTN | 0.361*** | 0.267** | 0.306** | 0.381*** | 0.294** | 0.362*** |
| SSD | 0.890*** | 0.958*** | 0.952*** | 0.913*** | 0.921*** | 0.851*** |

Note: RpH: pH of surface runoff; REC: Conductivity of surface runoff; SSR: surface runoff; ROC: Organic matter in surface runoff; RNN: nitrate nitrogen in surface runoff; RAN: ammonia nitrogen in surface runoff; RSP: soluble phosphorus in surface runoff; RTP: total phosphorus in surface runoff; RTN: total nitrogen in surface runoff; LpH: pH of interflow; LEC: Conductivity of interflow; SSL: interflow; LOC: Organic matter in interflow; LNN: nitrate nitrogen in interflow; LAN: ammonia nitrogen in interflow; LSP: soluble phosphorus in interflow; LTP: total phosphorus in interflow; LTN: total nitrogen in interflow. SSD: sediment yield. *: *p* < 0.05; **: *p* < 0.01; ***: *p* < 0.001. TCr, TCd, TCu, TNi, TPb and TZn represent the total amount of Cr, Cd, Cu, Ni, Pb and Zn in surface runoff, soil flow and sediment, respectively.

**Table S2.** Basic properties of the SS and soil.

| Variables | Sewage sludge | Soil |
| --- | --- | --- |
| pH | 9.33±0.05 | 5.00±0.07 |
| Organic matter (g·kg^-1^) | 204.79±1.14 | 14.71±0.35 |
| Cu (mg·kg^-1^) | 114.97±0.77 | 54.12±1.60 |
| Zn (mg·kg^-1^) | 475.55±6.91 | 120.55±8.88 |
| Pb (mg·kg^-1^) | 41.03±1.79 | 60.46±2.11 |
| Cr (mg·kg^-1^) | 94.40±4.53 | 72.90±2.53 |
| Ni (mg·kg^-1^) | 39.05±1.31 | 18.96±0.07 |
| Cd (mg·kg^-1^) | 1.89±0.12 | 0.58±0.08 |

**Table S3.** Experimental design.

| Indoor simulated rainfall experiment | | |  | Natural rainfall experiment | | |
| --- | --- | --- | --- | --- | --- | --- |
| Treatment | Slope (°) | Amount of SS applied (kg) |  | Treatment | Slope (°) | Amount of SS applied (kg) |
| CK | 15 | 0.00 |  | CK-F | 15 | 0.00 |
| S5 | 5 | 1.80 |  | S7-F | 7 | 180.72 |
| S15 | 15 | 1.87 |  | S15-F | 15 | 186.30 |
| S25 | 25 | 1.98 |  | S23-F | 23 | 198.54 |
